# Early detection of doxorubicin myocardial injury by ultrasonic tissue characterization in an experimental animal model

**DOI:** 10.1186/1476-7120-10-40

**Published:** 2012-10-10

**Authors:** Minna Moreira Dias Romano, Antônio Pazin-Filho, João Lucas O’Connel, Marcus Vinícius Simões, André Schmidt, Érica C Campos, Marcos Rossi, Benedito Carlos Maciel

**Affiliations:** 1 Divisions of Cardiology, Medical School of Ribeirão Preto, University of São Paulo, São Paulo, Brazil; 2 Divisions of Cardiology and Clinical Emergencies, Medical School of Ribeirão Preto, University of São Paulo, São Paulo, Brazil; 3 Department of Internal Medicine, Departament of Pathology, Medical School of Ribeirão Preto, University of São Paulo, São Paulo, Brazil; 4 Division of Cardiology, Department of Internal Medicine, Medical School of Ribeirão Preto, 14048-900, Ribeirão Preto, SP, Brazil

**Keywords:** Doxorubicin, Ultra-sonic tissue characterization, Echocardiography, Rats

## Abstract

In the clinical setting, the early detection of myocardial injury induced by doxorubicin (DXR) is still considered a challenge. To assess whether ultrasonic tissue characterization (UTC) can identify early DXR-related myocardial lesions and their correlation with collagen myocardial percentages, we studied 60 rats at basal status and prospectively after 2mg/Kg/week DXR endovenous infusion. Echocardiographic examinations were conducted at baseline and at 8,10,12,14 and 16 mg/Kg DXR cumulative dose. The left ventricle ejection fraction (LVEF), shortening fraction (SF), and the UTC indices: corrected coefficient of integrated backscatter (IBS) (tissue IBS intensity/ phantom IBS intensity) (CC-IBS) and the cyclic variation magnitude of this intensity curve (MCV) were measured. The variation of each parameter of study through DXR dose was expressed by the average and standard error at specific DXR dosages and those at baseline. The collagen percent (%) was calculated in six control group animals and 24 DXR group animals. CC-IBS increased (1.29±0.27 x 1.1±0.26-basal; p=0.005) and MCV decreased (9.1± 2.8 x 11.02±2.6-basal; p=0.006) from 8 mg/Kg to 16mg/Kg DXR. LVEF presented only a slight but significant decrease (80.4±6.9% x 85.3±6.9%-basal, p=0.005) from 8 mg/Kg to 16 mg/Kg DXR. CC-IBS was 72.2% sensitive and 83.3% specific to detect collagen deposition of 4.24%(AUC=0.76). LVEF was not accurate to detect initial collagen deposition (AUC=0.54). In conclusion: UTC was able to early identify the DXR myocardial lesion when compared to LVEF, showing good accuracy to detect the initial collagen deposition in this experimental animal model.

## Introduction

Cardiotoxicity is one of the most feared complications related to doxorubicin (DXR) prescription in clinical practice, expressed primarily by myocardial focal sclerosis, which can evolve to irreversible ventricular diastolic and systolic dysfunctions.

Serial quantification of left ventricle ejection fraction (LVEF) is the standard procedure to identify cardiotoxicity [1], but it is not sensitive enough to detect initial myocardial lesion [2,3]. Information regarding myocardial ultra-structure and composition, including the collagen deposition, can be provided by ultrasonic tissue characterization (UTC), a non-invasive technique that can detect and quantify acoustic properties of myocardial tissue [4,5]. Even though UTC has been used to detect myocardial damage in several myocardial collagen deposition diseases [6-10], its ability to do so in DXR cardiotoxicity is still debated. There is evidence of UTC abnormalities at the end of the DXR infusion [11-13]. In a previous pilot study performed in our laboratory (results yet not published), with rats receiving intraperitoneal DXR infusions, we found that UTC can potentially detect the myocardium texture alterations at the end of treatment with DXR, even in occasions where there was only a slight decrease in LVEF.

This study was designed to assess the capability of UTC to detect myocardium injury due to DXR infusion earlier than LVEF, as evaluated using echocardiography in an experimental animal model and with histological quantification of collagen deposition.

## Methods

### Study population

Sixty adults Wistar male rats (250 to 300g) were sedated and evaluated with echocardiographic examinations at basal situation as previously described [14]. Then, the animals received weekly endovenous DXR infusions of 2mg/Kg DXR up to a cumulative dosage of 16 mg/Kg. The animals were sedated and submitted to echocardiographic evaluations 7–15 days after the cumulative dosages of 8, 10, 12, 14, and 16 mg/Kg DXR. The protocol was approved by the Ethical Animal Research Committee of our institution, according to the Brazilian College Rules in Animal Experimentation (Protocol 041/2005).

### Two-dimensional conventional echocardiography

Two-dimensional echocardiography was performed, using an ultra-sound Sonos 5500, Philips (Andover, MA) equipment with S12-MHz sectorial probe. The parasternal short axis view at the papillary level was used to obtain chamber dimensions according to the M Mode technique — diastolic interventricular septal (IVS) and posterior wall (PW) thickness, end diastolic (LVEDD) and end systolic left ventricular diameters (LVESD). M-Mode images were obtained in a sweep speed of 100–150 mm/s what seems to have an adequate frame rate to an animal with a heart rate of 300bpm. These parameters were used to calculate the ejection fraction (EF) by Teichholz method [15] and shortening fraction (SF). Heart rate was measured with pulsed Doppler and all measurements were expressed as the average of three consecutive cardiac cycles.

### Ultrasonic tissue characterization

**Figure 1:**
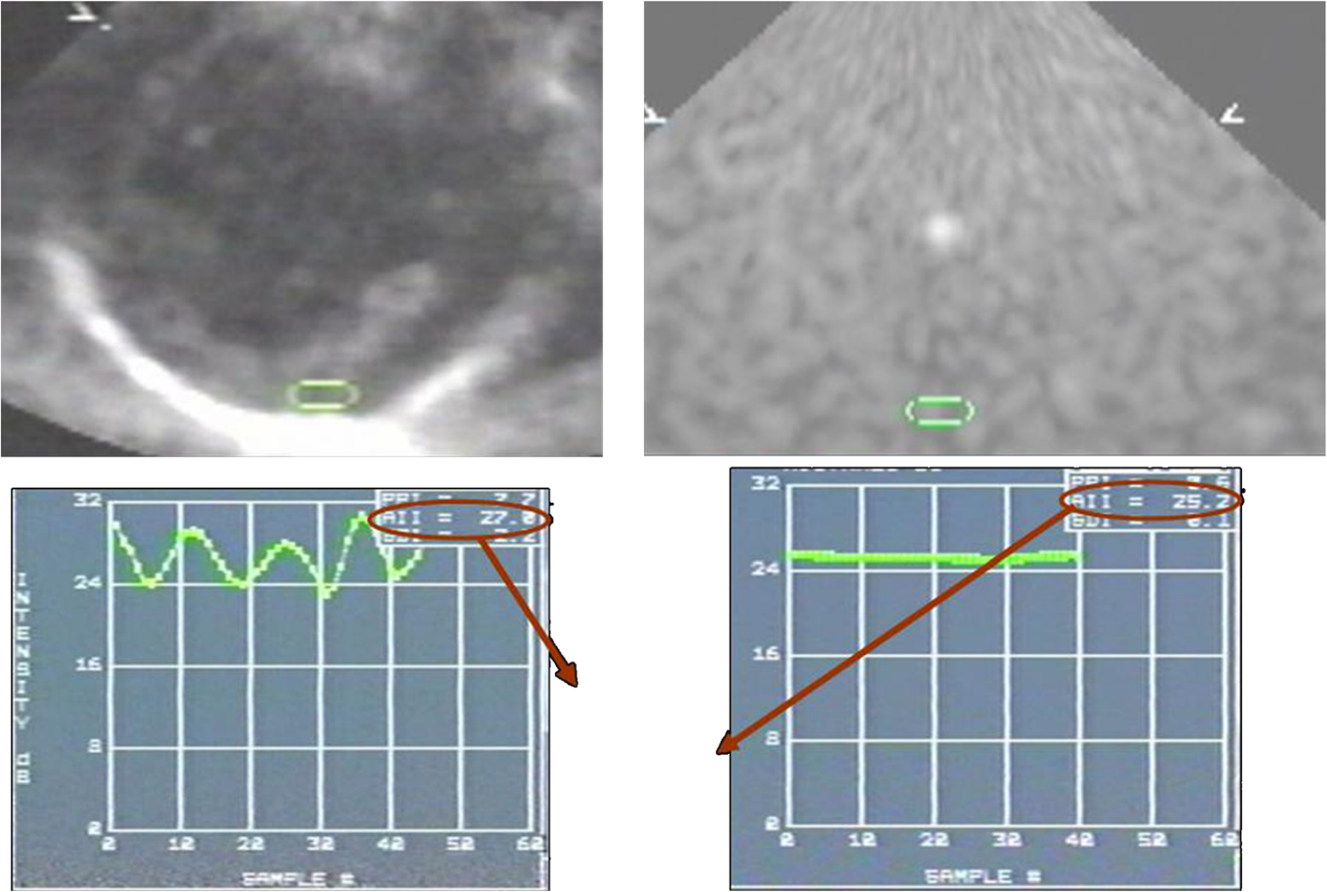
**Technical aspects of CC-IBS acquisition.** Upper left: parasternal view and sample collection of AII (Intensity signal) values of myocardium; lower left: AII curve of signals emanating from tissue; Upper right: sample collection of AII values from a rubber phantom at same depth, maintaining the same equipment adjustments; Lower right: AII curve signals emanating from the rubber phantom.

Two-dimensional ultrasonic backscatter images were obtained using an online acoustic densitometry package, incorporated in the same image system, from a parasternal short axis view at the papillary muscle level. Images were captured in a cineloop, displayed and stored for off-line analysis as follows: Backscatter (IBS) was measured from an elliptical sample placed over the posterior myocardium wall and made as large as possible, avoiding the epicardium and endocardium. The IBS values obtained were calibrated for system settings and for attenuation, by dividing the average value by the IBS value obtained from a rubber phantom (ATS Laboratories, 15 × 15 × 15cm, 15 db) for each segment, at the same depth. The IBS values of the phantom were obtained from backscattered echoes emanating from within the phantom at the same depth of the tissue that was analyzed (Figure 1). This variable was nominated as the corrected coefficient of the IBS (CC IBS). A second variable extracted from the IBS measurements was the magnitude of cardiac cyclic variation (MCV) of the IBS, as measured by the peak-to-peak intensity (PPI) difference between maximal and minimal values of IBS in the cardiac cycle.

### Histological analysis

Six control group animals, eight DXR group animals with cumulative dosage of 8 mg/Kg, six DXR group animals with cumulative dosage of 12 mg/Kg and ten DXR group animals with cumulative dosage of 16 mg/Kg were sacrified after sedation and submitted to histological analyses.; whole hearts were excised, fixed, and cross-sectionally cut at the papillary muscle level. Samples were colored with hematoxilin-eosin and red picrosirius, this last one for the collagen quantification. For each heart, ten randomly selected areas through the whole left ventricular wall, excepting the septum, were analyzed. Collagen was quantified with the Leica Qwin Software V 3.2.0 (Leica Imaging Systems Ltd., Cambridge, England) incorporated into an optical Leica DMR microscope (Leica Mycrosystems Wetzlar GmbH, Wetzlar, England), a video camera (Leica DC300F, Leica Mycrosysrems AG, Heerbrugg, Switzerland) and a computer station. Collagen amount was expressed as percentage of the measured area.

### Statistical analysis

Categorical variables were compared among all the groups with Fishers’ or Chi-Square tests and continuous variables with Kruskal-Wallis tests. To eliminate the effect of repeated measurements in the same animal, linear regression coefficient (beta coefficient) was obtained for each animal, utilizing the dependent variable and each DXR cumulative doses. Spearman test was used to evaluate the relation between beta coefficients and collagen percentage values. The receiver-operating (ROC) curve was elaborated according to a 4.24% collagen content (higher than the average value of the control group, plus 2 standard deviations) to determine the accuracy of both CC-IBS and left ventricular ejection fraction. All analyses were performed with STATA Intercool versão 9.2 (Stata Satistical Software 9,2 [computer program]. College Station TX StataComp; 2005).

## Results

Sixty animals were analyzed at baseline. Table 1 presents the DXR group data at basal and at DXR cumulative dosages of 8, 10, 12, 14 and 16 mg/Kg expressed as average and standard error values.

**Table 1:** DXR group data at basal and at DXR cumulative dosages of 8, 10, 12, 14 and 16 mg/Kg expressed in average and standard error values

**DOXORUBICIN**						
DOSE	0	8	10	12	14	16
N	60	23	21	14	9	7
MCV	11.0 ±2.6	9.1 ±2.8	8.4 ±2.3	8.2± 2	7.2 ±1.5	8.8 ±0.6
CC IBS	1.1 ±0.3	1.3± 0.27	1.2 ±0.1	1.4± 0.2	1.3 ±0.2	1.4 ±0.1
LVEF (%)	85.3± 6.9	80.4± 6.9	81.3± 5.6	76.4± 6.6	75.6± 4.8	67.3± 10
SF (%)	50±7.3	45.2 ±6.9	45.3 ±5.4	40.8 ±6.4	39.9 ±4.2	33.4± 7
Col (%)	1.7 ± 0.6	5.6± 1.6		10.6 ±2.5		9.6± 0.7
	(N=6)*	(N=8) *		(N=6)*		(N=10)*

N=animal number; MCV= magnitude of cyclic variation of IBS; CC IBS= corrected coefficient of IBS; EF Teic= Left ventricle ejection fraction (Teichholz); SF=left ventricle shortening fraction; Col=collagen percentage.* number of animals submitted to collagen analysis.

**Figure 2:**
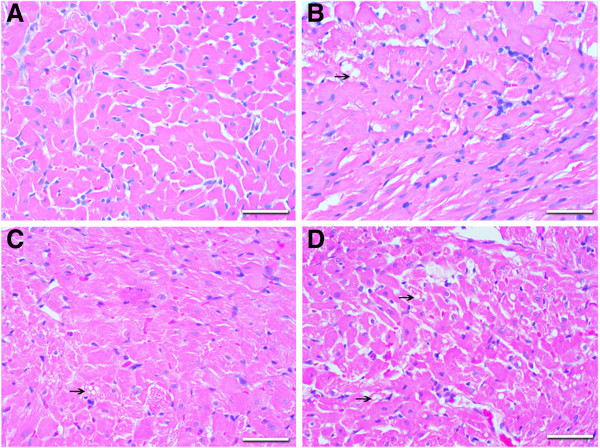
**Hystopathologic analysis of myocardial samples stained with hematoxilin and eosin from control (panel A) and doxorubicin groups (8 mg/Kg- panel B; 12 mg/Kg- panel C and 16 mg/Kg- panel D).** Note that vacuolar degeneration, disorganization and loss of myofibrils are more pronounced with the increment of DXR cumulative doses (panel **B**-**D**). Arrows mark points of vacuolar degeneration and intracellular edema.

The microscopic study of the myocardium revealed that the control hearts did not present any pathological change. The microscopic findings in the myocardium in all doxorubicin-treated groups were described as intracellular edema, vacuolar degeneration, and disorganization or loss of myofibrils. Loss of myofibrils and vacuolar degeneration of cardiomyocytes were predominantly characterized by alterations in the doxorubicin cardiomyopathy, more pronounced in the myocardium of the doxorubicin groups treated with 12 and 16 mg/kg (Figure 2).

**Figure 3:**
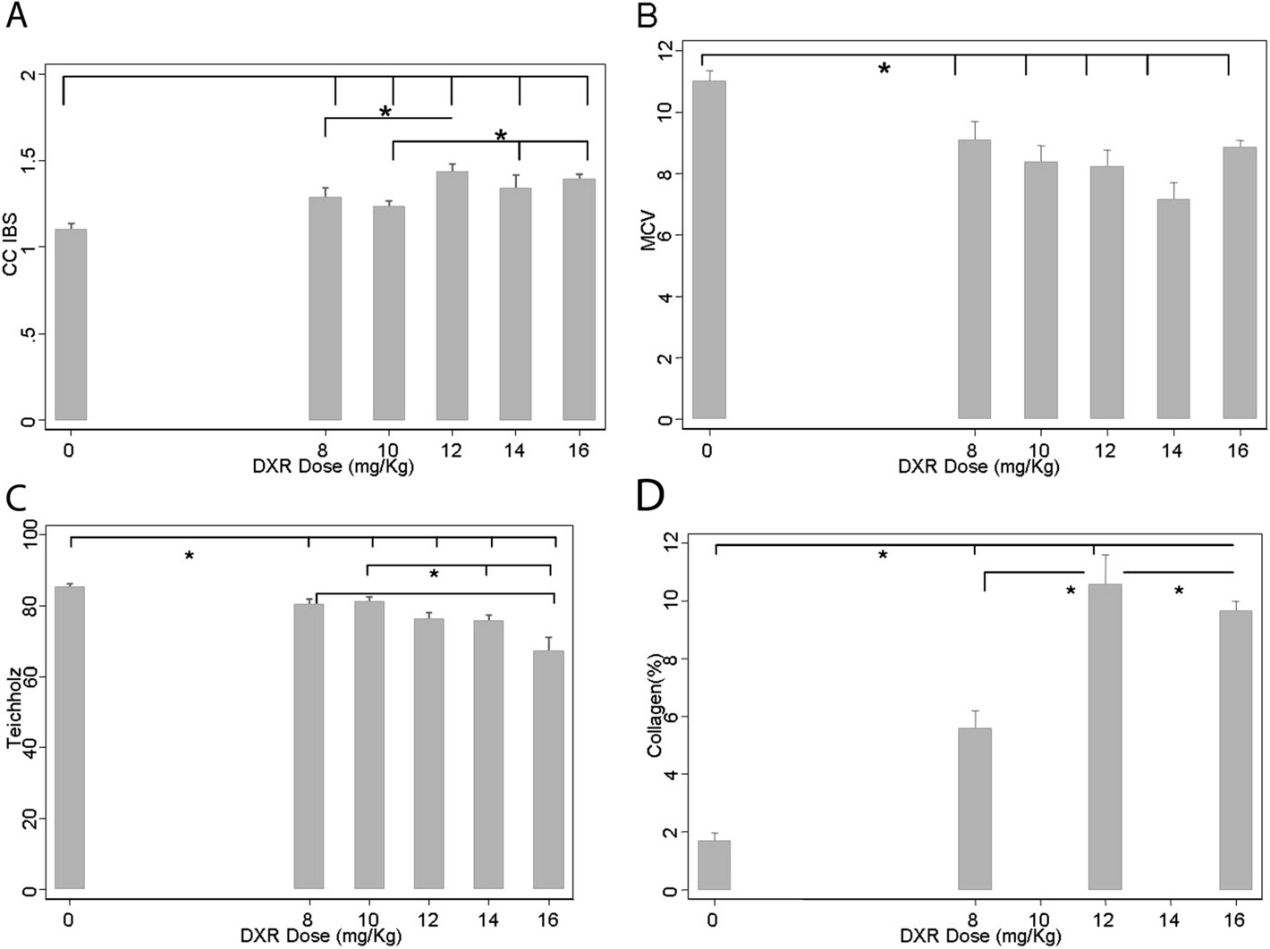
**Average values of CC-IBS (panel A), magnitude of IBS cyclic variation- MCV (panel B) and LVEF by Teichholz method (panel C) throughout cumulative DXR dosages in basal and DXR treated animals.** Panel **D** shows collagen percent detected in each DXR cumulative dosages groups. *=p<0.05 between dosages.

**Figure 4:**
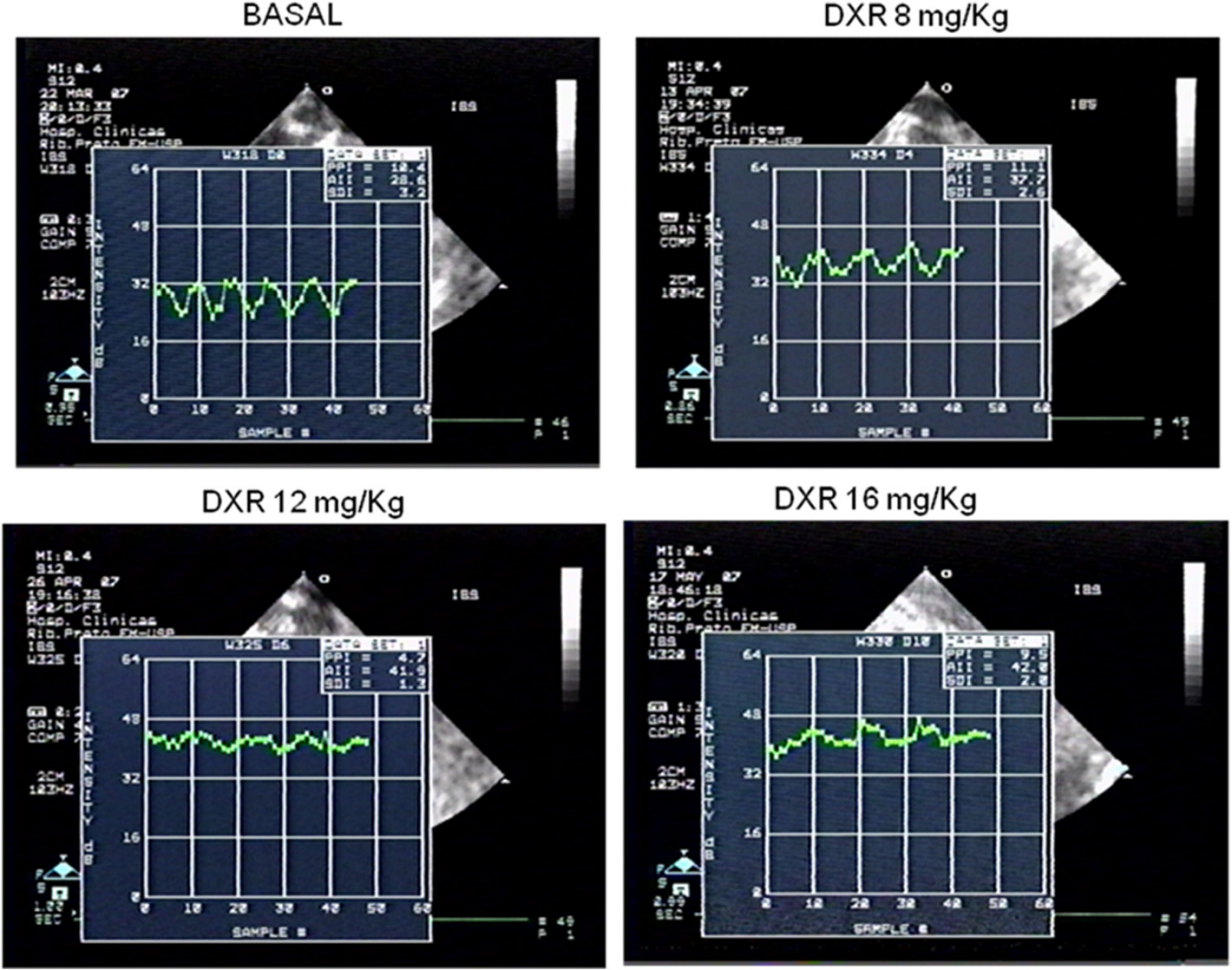
Examples of IBS intensity values from rats of different cumulative DXR dosages.

**Figure 5:**
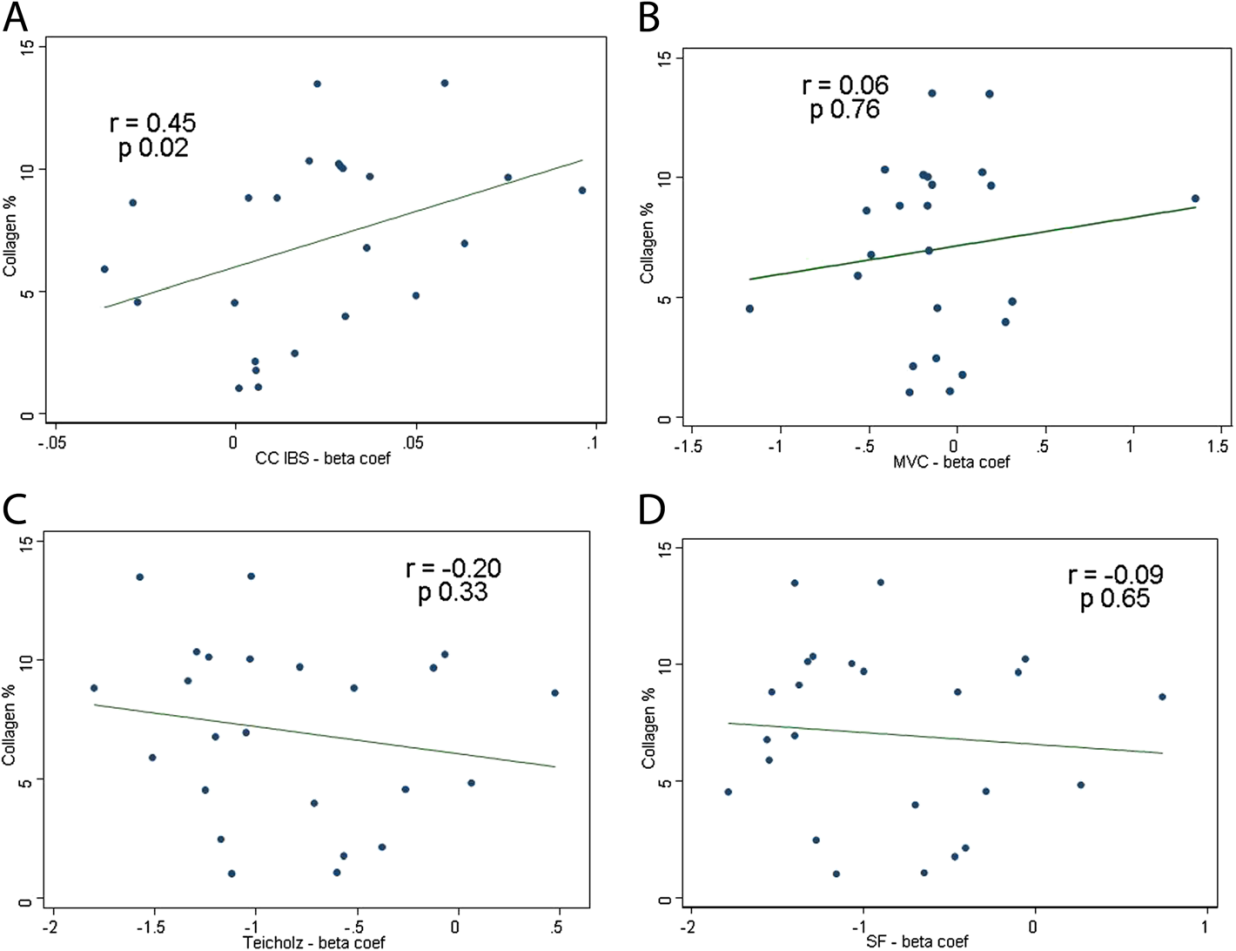
**Correlation analysis between collagen content (%) and beta coefficient of: CC-IBS (panel A); magnitude of IBS cyclic variation -MCV (panel B); LVEF using Teichholz formula (panel C) and SF (panel D).** r= Spearman correlation coefficient and e p significative <0,05.

Collagen content increased through DXR infusion from 8 mg/Kg (5.6±1.6% x 1.7±0.6-basal; p=0.001) (Figure 3, panel D). With each DXR incremental dose, there was a continuous increase of CC-IBS and decrease in LVEF (Figure 3-panels A and C). CC-IBS increased significantly with a DXR cumulative dosage of 8 mg/Kg (1.29±0.27 x 1.1±0.26-basal; p=0.005), with a subsequent increase at 12 mg/Kg dosage (1.4±0.2 x 1.1±0.26-basal p=0.005); while MCV decreased from 8 mg/Kg DXR dosage (9.1± 2.8 x 11.02±2.6-basal; p=0,006) (Figure 3-panel B) and remained stable after. The decrease in LVEF at DXR cumulative dosage of 8 mg/Kg was lower than 6% (80.4±6.9% x 85.3±6.9%-basal, p=0.005). At the same cumulative dose (8 mg/Kg DXR), CC-IBS increased nearly 20% from basal values, reaching a 30-40% increment at 12mg/Kg DXR (Figure 3- panels A and C). Figure 4 illustrates actual ultrasonic findings, with IBS calculations from animals in different DXR dosages. There was a significant correlation between collagen content and beta coefficient of CC-IBS (r=0.45, p=0.02) (Figure 5 – panel A) but no correlation was observed between collagen content and MCV (r= 0.06, p=0.76) (Figure 5-panel B), LVEF- Teichholz (r= −0.20, p=0.33) (Figure 5- panel C), and SF (r=−0.09, p=0.65) (Figure 5- panel D).

**Figure 6:**
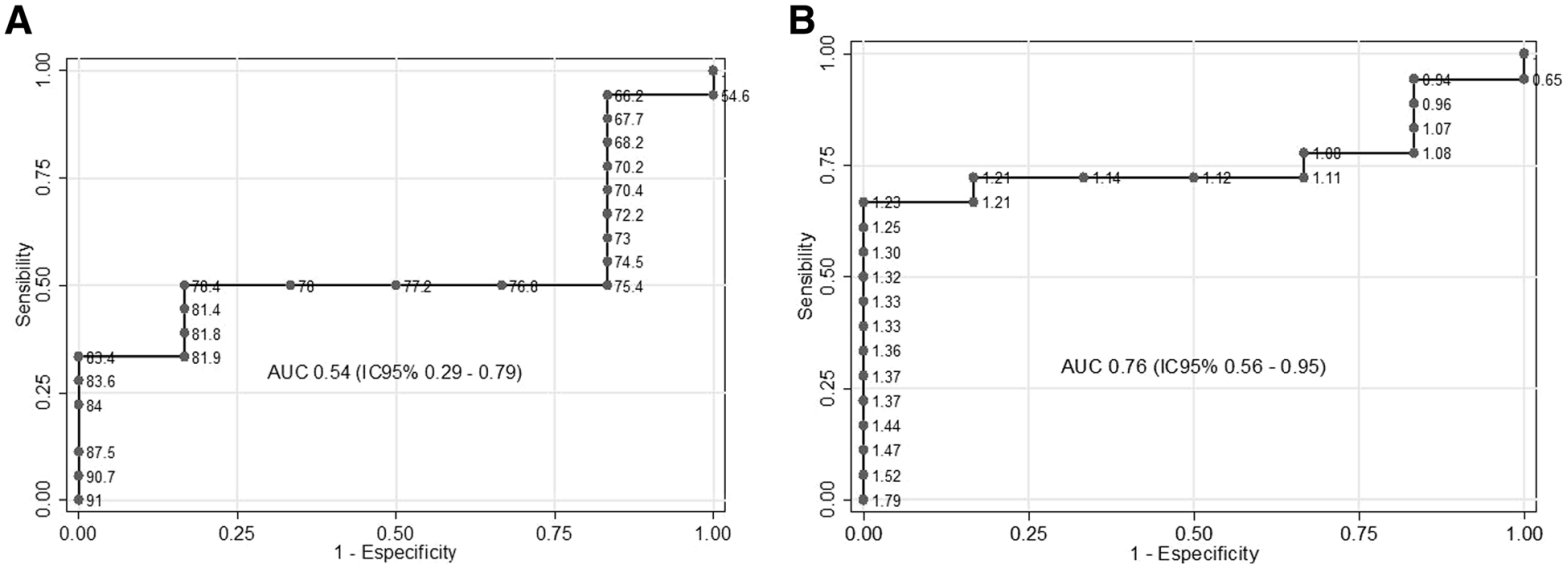
**Lef ventricle ejection fraction accuracy analysis (Panel A) and CC-IBS (Panel B) to detect a 4.24% myocardial collagen content.** ROC= Receive-operating curve; AUC= area under the curve; IC= confident interval.

LV ejection fraction was not accurate enough to detect initial myocardium collagen deposition (4.24% of myocardial content) (AUC=0.54) (Figure 6-panel A). CC-IBS was able to detect the collagen deposition induced by DXR, with good accuracy (AUC=0.76). A CC-IBS value of 1.21 was able to detect initial collagen deposition (4.24% of myocardial content) with a sensitivity of 72.2% and specificity of 83.3% (Figure 6-panel B).

## Discussion

To the best of our knowledge this is the first evidence that UTC, by using CC-IBS, can detect the initial myocardial lesion (collagen deposition) secondary to DXR infusion early than clinical significant LVEF decrease. Our results are congruent with previously published data [16] showing that this experimental animal model of DXR infusion reproduces pathologic myocardial alterations similar to those found in human disease.

At the end of the DXR infusion protocol, LVEF decrease was about only 6% at 8 mg/Kg DXR and about 10% at 12 mg/Kg. This result is in agreement with previously published data showing that the LVEF decreases slightly at 12 weeks of DXR infusion [17-20].

Information regarding myocardial ultra-structure and composition can be provided by ultrasonic tissue characterization, a non-invasive technique capable of detecting and quantifying acoustic properties of myocardial tissue (4;5). The IBS intensity is related to physical and structural properties of the myocardium and is particularly influenced by tissue collagen content and spatial distribution of this component [21]. In addition, IBS intensity also depends on ultrasound system settings and on ultrasound attenuation, which has been a major limitation for the current use of this index. In order to overcome this limitation, several methods have been employed to calibrate the absolute value of IBS according to a reference value, derived from the ventricular cavity [22], the pericardium [7] or from a gray scale phantom [23][13] such as the one used in this investigation. Although absolute IBS values derived from ventricular cavity or pericardium could reduce the influence of the ultrasound system settings, there are limitations for their use so as to correct the effects of ultrasound attenuation; This, associated to the fact that the pericardium membrane (and consequently its IBS intensity) could be affected by DXR toxicity, influenced our choice of a rubber phantom referential for this investigation.

There is little published data about CC-IBS in this animal model, but Mimbs, studying rabbits, has already demonstrated CC-IBS increments at the end of the total cumulative dose of DXR infusion. In this study we reproduced their findings, but we were also able to demonstrate a dose–response relationship beginning at an 8mg/Kg cumulative dose. Also, CC-IBS was the only index with a significant correlation with collagen deposition, secondary to DXR infusion. The capability of IBS in detecting fibrosis content of the myocardium was already described in several diseases, including dilated cardiomyopathy [22,24].

During cardiac contraction and relaxation, a cardiac cycle-dependent variation of myocardial IBS can be documented. The magnitude of cyclic variation of the IBS, measured as peak-to-peak variation of the IBS curve (MCV), is an additional acoustic parameter, independent on ultrasound system settings and responsible for its wider application. However, this index is more strictly correlated with the myocardial contractile function than the collagen myocardial content. Previously published data showed a decrease in MCV with DXR infusion [11,20,25], even when LVEF was preserved. However, Ha et al., using the same experimental animal model described in our study, failed to demonstrate the decrease in MCV with DXR infusion [17]. Considering that our investigation evaluated MCV through the whole infusion DXR protocol, our results are the first to demonstrate that MCV decrease occurs at initial cumulative DXR dosages (8mg/Kg). MCV of IBS could be used, in addition to CC-IBS, to detect early myocardial DXR toxicity. MCV of IBS may be more related to the myocardium contractile function than collagen content. If so, the reduction of MCV from initial dosages of DXR infusion may accompany initial contraction impairment, as suggested in recent studies [26-29] using regional contraction analysis techniques like “strain rate”. Maybe cellular loss, which is the initial myocardium damage before full collagen deposition, could be detected with these tools (MCV of IBS and by myocardium deformation analysis data, like strain, strain rate and speckle tracking techniques) in a similar way.

The good accuracy of CC-IBS, as demonstrated here, suggests that this parameter could be a non-invasive tool to detect initial collagen deposition secondary to DXR infusion, and it may have the potential to be used as an alternative tool for serial evaluation of myocardial lesions in the clinical setting.

**Clinical applicability**: this work confirms the capability of UTC using CC-IBS to detect the initial myocardial DXR lesion earlier than the decrease of LVEF in rats. The UTC technique using the CC-IBS is feasible in serial myocardial assessments in humans. As such, this study brings a perspective in the use of UTC as a non-invasive method in collagen myocardium detection, and as an alternative tool for endomyocardial biopsies, with good accuracy, but human studies are needed.

**Limitations**: the anatomo-pathological studies were limited to the rats that completed the protocol phase and which had not suffered spontaneous deaths. In fact, if those who died were included more striking differences could have been obtained, but adding some confounding factors. The high mortality rates at final cumulative dosages of DXR limited the number of animals analyzed at this point. This study did not include other pathologic myocardium analysis methods, such as myocardial fibers diameter quantification, ultra-structural myocardial analysis, or analysis of the tridimensional myocardial arrangement that could be better correlated to UTC indices like MCV. Another significant limitation is the lack of diastolic data. Since our acoustic window was limited and did not permit a collection of distinct validated diastolic dysfunction parameters, we preffer to exclude it from the protocol. A specific protocol can be developed from this work to address this issue. In summary, UTC was able to identify the DXR myocardial lesion earlier when compared to LVEF, showing good accuracy to detect the initial collagen deposition in this experimental animal model.

## Competing interests

The authors declare that they have no competing interest.

## Authors’ contributions

MMDR: acquisition of data, analysis and interpretation of IBS and data drafted the article. APF: revised it critically for important intellectual content. JLO: participated in the conception of the study and acquisition of data. MVS: **substantial contributions to conception** and design. AS: helped to draft the article and revised it critically for important intellectual content. ECC: participated in the acquisition of anatomo-pathological data, its analysis and interpretation. MR: gave important contributions to analysis and interpretation of anatomo-pathologic data. BCM: conceived the study and revised it critically for important intellectual content. All authors read and approved the final manuscript.
